# Translating sickle cell guidelines into practice for primary care providers with Project ECHO

**DOI:** 10.3402/meo.v21.33616

**Published:** 2016-11-24

**Authors:** Lisa M. Shook, Christina B. Farrell, Karen A. Kalinyak, Stephen C. Nelson, Brandon M. Hardesty, Angeli G. Rampersad, Kay L. Saving, Wanda J. Whitten-Shurney, Julie A. Panepinto, Russell E. Ware, Lori E. Crosby

**Affiliations:** 1 Department of Pediatrics, Cincinnati Comprehensive Sickle Cell Center, Division of Hematology, Cancer and Blood Diseases Institute, Cincinnati Children's Hospital Medical Center, Cincinnati, OH, USA; 2 Children's Hospitals and Clinics, Minneapolis, MN, USA; 3 Indiana Hemophilia and Thrombosis Center, Inc., Indianapolis, IN, USA; 4 Children's Hospital of Illinois, OSF Saint Francis Medical Center, University of Illinois College of Medicine, Peoria, IL, USA; 5 Sickle Cell Disease Association of America, Michigan Chapter, Detroit, MI; 6 Children's Hospital of Wisconsin, Medical College of Wisconsin, Milwaukee, WI, USA; 7 Division of Behavioral Medicine, Department of Pediatrics, Cincinnati Children's Hospital Medical Center, Cincinnati, OH, USA

**Keywords:** provider education, telementoring, continuing education, sickle cell disease

## Abstract

**Background:**

Approximately 100,000 persons with sickle cell disease (SCD) live in the United States, including 15,000 in the Midwest. Unfortunately, many patients experience poor health outcomes due to limited access to primary care providers (PCPs) who are prepared to deliver evidence-based SCD care. Sickle Treatment and Outcomes Research in the Midwest (STORM) is a regional network established to improve care and outcomes for individuals with SCD living in Indiana, Illinois, Michigan, Minnesota, Ohio, and Wisconsin.

**Methods:**

STORM investigators hypothesized that Project ECHO^®^ methodology could be replicated to create a low-cost, high-impact intervention to train PCPs in evidence-based care for pediatric and young adult patients with SCD in the Midwest, called STORM TeleECHO. This approach utilizes video technology for monthly telementoring clinics consisting of didactic and case-based presentations focused on the National Heart, Lung and Blood Institute (NHLBI) evidence-based guidelines for SCD.

**Results:**

Network leads in each of the STORM states assisted with developing the curriculum and are recruiting providers for monthly clinics. To assess STORM TeleECHO feasibility and acceptability, monthly attendance and satisfaction data are collected. Changes in self-reported knowledge, comfort, and practice patterns will be compared with pre-participation, and 6 and 12 months after participation.

**Conclusions:**

STORM TeleECHO has the potential to increase implementation of the NHLBI evidence-based guidelines, especially increased use of hydroxyurea, resulting in improvements in the quality of care and outcomes for children and young adults with SCD. This model could be replicated in other pediatric chronic illness conditions to improve PCP knowledge and confidence in delivering evidence-based care.

Sickle cell disease (SCD) is a group of disorders, in which red blood cells change to a ‘sickled’ crescent shape, which leads to a myriad of acute and chronic clinical complications. SCD affects millions of people around the globe, and is frequently found among persons living in North and South America, the Caribbean and Central America, India, Saudi Arabia, and Mediterranean countries (Turkey, Greece, and Italy) (1).

SCD is among the most common disorders identified via universal newborn screening in the United States, with an incidence higher than that for all other genetic conditions including congenital hypothyroidism and cystic fibrosis (2–5). There are approximately 100,000 patients living with SCD in the United States (6), including an estimated 15,000 patients located in the Midwest (7).

SCD was once primarily a childhood disease, since many patients died before adulthood; today over 95% of children diagnosed at birth survive to young adulthood but still suffer early mortality by age 40 years (8, 9). This shift in mortality from childhood to adulthood has been accompanied by an increase in serious morbidity from chronic organ damage among teens and young adults including cerebrovascular, cardiopulmonary, and renal disease, all of which are major contributors to early death (10, 11). Severe pain is the most common cause for hospitalization and acute morbidity, and requires extensive pain management that patients receive from various clinical settings, including Emergency Departments and primary care providers (PCPs) (12).

In late 2014, the National Heart, Lung and Blood Institute (NHLBI) of the National Institutes of Health published evidence-based guidelines for the management of pediatric and adult SCD, including recommendations for increasing hydroxyurea use in all age groups (13). Hydroxyurea is a daily oral medication that can change the clinical course of SCD, with significant reduction in the frequency of hospitalizations, transfusions, pain, and other complications, (14–16) and increased survival for both pediatric and adult patients (17–20). Despite these known clinical benefits, only 20–30% of eligible adults in the United States are prescribed the medication (21, 22). Provider knowledge and comfort level with hydroxyurea is one of the barriers that have limited access to and use of the therapy by patients (23). Many provider concerns about long-term side effects are based solely on theoretical risks; for example, studies have shown that 27% of pediatric providers (24) and 40% of adult providers (25) fear that patients could develop cancer from taking hydroxyurea, despite the substantial clinical experience to date that does not support those concerns (26, 27).

Complex chronic diseases such as SCD can be difficult to manage, especially for PCPs with limited experience, ancillary support, and time to manage SCD-related pain and complications (28). While specialists exist for pediatric care, there is a paucity of hematologists specializing in SCD for adults, which makes accessibility to PCPs knowledgeable about SCD vital. Unfortunately, there are few PCPs who are well-informed about the guidelines and confident in delivering evidence-based care to the SCD population. PCPs and family physicians generally report a low comfort level with treating patients with SCD, which is particularly true for providers who see few patients with SCD in their practice (29).

To increase the knowledge and comfort level of PCP's caring for children and adults with SCD in the Midwest, the Sickle Treatment and Outcomes Research in the Midwest (STORM) regional network replicated the Project ECHO^®^ model. Project ECHO (Extension for Community Healthcare Outcomes), developed at the University of New Mexico (UNM) Health Sciences Center in 2003 (30), is an innovative approach to building PCPs’ capacity to treat complex diseases by giving them specialized knowledge and support (31). The four principles of Project ECHO are 1) using telehealth technology to build healthcare resources where they are scarce, 2) sharing best practices to reduce variation in clinical care, 3) utilizing practice-based learning to develop specialty expertise among providers, and 4) monitoring and evaluating provider outcomes (32). Project ECHO was primarily designed for rural outreach to providers, but several projects have demonstrated their effectiveness in an urban setting (33).

We hypothesize that Project ECHO could be replicated to provide specific training for PCPs caring for children and adults with SCD, and promote effectively prescribing hydroxyurea, ultimately resulting in improved clinical care and outcomes. We now describe our methods and approach for replicating the Project ECHO model, including the outcomes targeted to assess its effectiveness at increasing PCP knowledge, competence, and confidence, and ultimately changing practice behaviors in managing patients with SCD.

This continuing education model documents provider attendance in the continuing medical education (CME) intervention and also follows an interactive, expanded outcomes educational framework with an emphasis on educational planning and instructional design throughout the activity, to ultimately change provider competence and performance.

## Methods

### STORM TeleECHO

STORM is a regional network funded by the Health Resources and Services Administration (HRSA) as a Sickle Cell Disease Treatment Demonstration Project. The grant is based at Cincinnati Children's Hospital Medical Center (CCHMC) and covers the states of Indiana, Illinois, Michigan, Minnesota, Ohio, and Wisconsin. STORM seeks to increase the number of PCPs knowledgeable about the overall management and treatment of SCD, and especially to increase the number prescribing hydroxyurea.

The STORM TeleECHO consists of a ‘hub’ team that is led by the STORM Regional Coordinating Center, based at the Cincinnati Comprehensive Sickle Cell Center within CCHMC. The hub team includes a multidisciplinary clinical expert team, project management support, and technology support from the Center for TeleHealth. The STORM TeleECHO hub team was the first to participate in immersion training by the American Academy of Pediatrics (AAP) Superhub team in December 2015. The AAP Superhub team serves as a resource center that provides technical assistance and leverages resources for the STORM TeleECHO team from planning through implementation.

STORM Networks sites (one lead in each state) and PCP sites (numerous locations in each state) serve as STORM TeleECHO ‘spokes’. STORM Network sites began recruiting PCPs in January 2016 via personal and professional connections, outreach at provider conferences, and mailings and emails to providers. To date, most spokes have recruited 3–4 provider groups with a goal of 10–20 per state. Provider groups include at least one physician, but may include a multidisciplinary team. Upon registering, providers are required to complete a baseline assessment about their practice and knowledge, comfort level, and experience treating patients with SCD, including hydroxyurea prescribing practices. This formative assessment is important to guide the educational content of STORM TeleECHO to meet the learners’ needs and education gaps, while aligning the curriculum with evidence-based care guidelines.

### STORM TeleECHO clinic design

The TeleECHO clinic model utilizes telementoring and state-of-the-art, low-cost technology to link subspecialists and PCPs. Telementoring is defined as the use of electronic information and telecommunication technologies to support long-distance professional health-related education (34). TeleECHO clinics can range from 60 to 120 min and include a brief 15–20 min didactic followed by 1–2 case presentations with facilitated discussion. STORM TeleECHO didactics topics were selected based on the NHLBI guidelines (13) and are presented by nationally recognized pediatric and adult hematologists with expertise in SCD (Table 1). Providers and other participants submit cases using a HIPAA-protected, de-identified pediatric or adult case-presentation template that includes relevant medical history, laboratory results, psychosocial history, and treatment plan to the ‘hub’ prior to clinics. To protect patient confidentiality, case presenters are instructed to refer to the case ID and template during their verbal presentations. The goal of the discussions is to promote learning through a robust discussion of clinical and psychosocial issues. During the discussion, all participants are permitted to ask questions and/or provide treatment recommendations. Audience participation and interaction is encouraged, and one strategy for facilitating a robust discussion is integrating an audience-response system to gauge clinical decisions during the case discussion. After the clinic, case presenters receive a written summary of the recommendations with the following disclaimer:

based upon general scientific principles, intended for broad and general physician understanding and knowledge and is offered solely for educational purposes, and should in no way constitute a formal or informal medical consultation.

**Table 1:** STORM TeleECHO didactic topics

NHLBI guidelines content	Additional evidence-based content
Acute chest syndrome	Abdominal complaints
Chronic pain	Emergency department use
Hydroxyurea	Health equity
Leg ulcers	Healthy living with SCD
Overview of adult complications	Home pain management plan
Overview of pediatric complications	Neurocognitive complications
Pain management	Newborn screening follow-up
Pediatric infection	Patient experience panel
Priapism	Psychosocial issues
Pulmonary complications	Quality of life
Renal complications	Transition to adult care
Retinopathy	Stroke
Transfusions	

NHLBI, National Heart, Lung and Blood Institute; SCD, sickle cell disease; STORM, Sickle Treatment and Outcomes Research in the Midwest.

All treatment decisions are therefore the responsibility of the presenter, and none of the STORM TeleECHO partners is liable for any adverse events. Presenters can then follow up with STORM sites in their respective states for localized mentoring, consultations, or referrals. Because the purpose of STORM TeleECHO is to improve the knowledge of regional PCPs, the CCHMC Institutional Review Board deemed it exempted from review and waived the requirement for written informed consent. Providers who wish to receive *AMA PRA Category 1* CME credits from CCHMC for attending the STORM TeleECHO clinics must complete a summative evaluation after each session, which includes an assessment to determine if the learning objectives presented will lead to the desired outcomes of changed practice behavior.

### Technology

STORM TeleECHO participants are encouraged to participate using the Internet and a webcam to create a virtual learning environment. The Cisco WebEx™ technology platform is used during the clinics. Didactic presentations are recorded and archived on a password-protected Sharepoint site, along with supplemental educational materials, that can be accessed by participants after the STORM TeleECHO clinics as reinforced learning activity tools.

## Results

### STORM performance

**Fig. 1:**
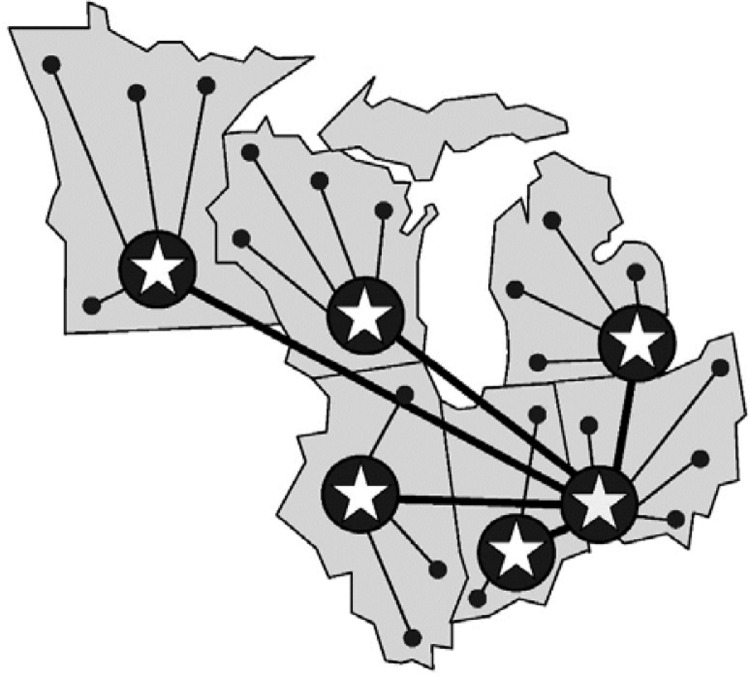
STORM regional network TeleECHO map. Six states, namely Ohio, Indiana, Illinois, Michigan, Minnesota, and Wisconsin, are involved in the network.

During the first 6 months of STORM TeleECHO, 6 monthly teleclinics have been conducted. There have been 38 registered participants from 7 states including all states in the STORM region (see Fig. 1), and 4 observers from federal agencies and professional organizations. Physicians (64%), nurse practitioners (5%), and other disciplines (31%) have participated in STORM TeleECHO. There have been an additional 26 participants who have attended clinics without registering, or registered but not attended clinics to date. Practice specialties include pediatric and adult hematology, internal medicine, family practice, and primary care. Didactic presentations include an overview of pediatric complications; an overview of adult complications in SCD; newborn screening for hemoglobin disorders; psychosocial issues in SCD; hydroxyurea; and abdominal complications. Nearly 100% of participants have reported an increase in knowledge about SCD management as a result of STORM TeleECHO.

### STORM sites and PCP outreach

**Fig. 2:**
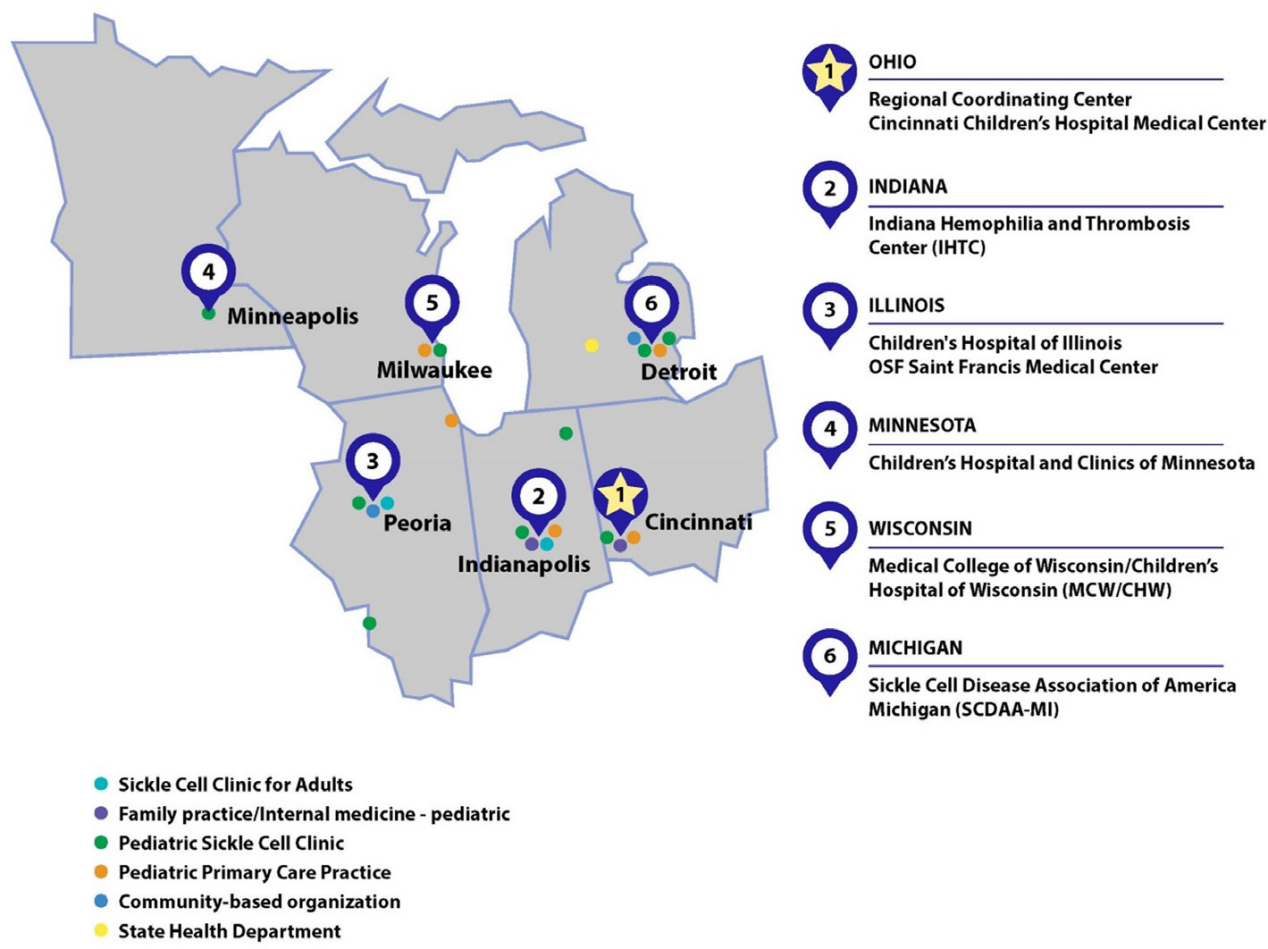
STORM regional network TeleECHO map of participants.

The geographic setup of the STORM network is illustrated in Fig. 2. All six states in the STORM network have a single physician champion and have recruited several PCPs/specialists who follow patients with SCD and have limited access to SCD resources. With an ongoing networking and a strategic marketing plan to engage more PCPs to join STORM TeleECHO clinics, the number of potential patients’ whose care will be impacted by transformed clinical practice will increase.

### Feasibility and acceptability

Monthly outcome measures include clinic attendance, attendee type (PCP, subspecialist, physician assistant, etc.), satisfaction, and number and type of cases presented. These data (except for satisfaction) are tracked via a Project ECHO secure web-based data platform, known as iECHO^TM^, which is a Project ECHO network project management tool that tracks attendance, number and type of case presentations, and the number of CME credits awarded to participants. No protected health information is entered into the database. Clinic evaluations to assess satisfaction follow standard wording required by the CCHMC Office of Continuing Medical Education.

### Effectiveness and impact

Provider knowledge and self-efficacy of the NHLBI SCD guidelines and practice patterns will be assessed at baseline, 6 and 12 months after participating in STORM TeleECHO clinics (Table 2). Evaluations are administered via a SurveyMonkey web-based survey after each monthly clinic. Surveys use Likert-scale and open-ended questions to assess clinical expertise, knowledge of evidence-based guidelines for SCD, and hydroxyurea prescribing patterns. Qualitative data on program effectiveness demonstrated by self-reported practice changes as an objective measure of impact will also be summarized and used to make program adaptations.

**Table 2:** STORM TeleECHO provider self-efficacy, benefits, and practice measures

Provider self-efficacy: rate your ability to do each of the following
Provide primary care for pediatric patients with SCD
Provide primary care for adult patients with SCD
Manage acute pain in pediatric patients with SCD
Manage acute pain in adult patients with SCD
Manage chronic pain in pediatric patients with SCD
Manage chronic pain in adult patients with SCD
Identify suitable candidates for disease modifying therapies, such as hydroxyurea
Prescribe disease modifying therapies, such as hydroxyurea
Serve as a provider for SCD patients
Provider benefits: provide your assessment for each of the following
Through the STORM TeleECHO telehealth clinics, I am learning best-practice care for SCD
I learn with guidance from STORM TeleECHO specialists in SCD management, whose knowledge and skills I respect
I am developing my clinical expertise through participation in STORM TeleECHO
My participation in STORM TeleECHO benefits patients under my care
The patients under my care receive best-practice care for SCD
I apply what I have learned about best practices through STORM TeleECHO to all of my patients with SCD
Provider practices: describe the current treatment patterns for your patients
Of the patients you see with SCD, what percentage is eligible for hydroxyurea?
Of the patients eligible, what percentage has been prescribed hydroxyurea?
What percentage of patients is up to date with pneumococcal vaccinations, as documented in the medical record?
What percentage of patients is on chronic transfusions (>4–6 transfusions in the past 12 months)?

## Discussion

Project ECHO was originally created to increase access to care for patients with hepatitis C in rural New Mexico because rural providers were unprepared to treat these patients. The UNM Health Sciences Center, the primary subspecialty clinic in the state, sought to reduce wait times for appointments, decrease travel for patients with hepatitis C, and improve treatment outcomes (35). They also hoped to build a bridge between rural PCPs and an urban academic medical center. UNM Project ECHO has since partnered to replicate the model in an effort to ‘demonopolize’ medical knowledge to improve healthcare in patients with diabetes, asthma, HIV/AIDS, chronic pain, pediatric obesity, substance use disorders, cardiovascular conditions, rheumatoid arthritis, and mental illness. Data consistently show that through participation in ECHOs, community-based PCPs are able to provide higher quality evidence-based chronic disease care and management (36). An additional benefit has been the creation of ‘communities of practice’, which increases PCP's satisfaction and reduces isolation and burnout (37).

This is the first report of adapting Project ECHO to patients with SCD. Our design has replicated the UNM Project ECHO model using a regional approach to translate the NHLBI's evidence-based sickle cell management guidelines to improve the practice of PCPs treating pediatric and young adult patients with SCD. The Project ECHO model allows providers to connect to academic medical centers and specialists in an interactive, performance-based continuing education forum that will improve their knowledge and comfort level, and change practice behaviors (38). In this way, the STORM TeleECHO project will help to achieve our STORM network goals of increasing the number of providers knowledgeable about SCD, and increasing the number of providers prescribing hydroxyurea. The data on attendance, participation (e.g., submitting cases), and overall satisfaction will provide information about the feasibility of this method for increasing provider self-efficacy in managing SCD in children and young adults.

There are many potential benefits to adapting the Project ECHO model for improving evidence-based care of SCD. Use of a low-cost technology for provider education should be beneficial particularly for providers who cannot attend conferences and meetings due to time limitations and travel costs, and especially for PCPs located in low-resource settings. Moreover, the model allows providers to participate in the educational activity in their own clinic setting, along with their local clinical team, thereby increasing the likelihood of application.

Further, the Project ECHO model is designed to facilitate meaningful, interactive, continuous learning. The model closely follows the framework of outcomes assessments in developing physician clinical skills. Utilizing the NHLBI evidence-based guidelines as the didactic presentation curriculum, providers have a guide for what declarative knowledge they *should* have when treating patients. Second, ‘knowing how’, or procedural knowledge, may be further refined for providers who may not be comfortable managing patients or prescribing hydroxyurea. Though the ‘community of practice’ and mentoring, providers begin to build the procedural skills of how to manage SCD by presenting cases, or participation in case discussions. Finally, providers can demonstrate their competency by implementing what they have learned through STORM TeleECHO and perform more confidently and improve patient outcomes (39) while having continuous feedback and mentoring from clinical experts.

Project ECHO's educational foundation is based on postgraduate medical education principles and grounded in three key learning theories: social cognitive theory, situated learning theory, and community of practice theory (40). Social cognitive theory is the idea that participants must believe there is a benefit in learning a new behavior, develop confidence in performing this behavior, and receive reinforcement of positive behavior changes (41). Situated learning theory centers on the concept of providing learners with modeled experiences to engage their interest and simplify tasks while learning a new skill (42). Community of practice theory focuses on the significance of participating in a collaborative community with peers and content experts, on a continual basis (43). A recent study of urban community health center providers in Chicago, who participated in an ECHO project to improve management of resistant hypertension, showed that the ECHO model effectively combines these key established learning theories to maximize educational opportunity for participants (36). STORM TeleECHO will build a community of practice for SCD in the Midwest. Regional PCPs, who may have had limited interactions with hematology specialists, will be able to build strong relationships within the network that will be beneficial for evidence-based care and co-management throughout the lifespan of patients with SCD: from initial diagnosis with newborn screening to the transition from teen to young adult care, and even to managing severe complications later in life.

This model for regional telementoring is not without limitations. The STORM TeleECHO project may initially reach only providers with a vested interest in caring for patients with SCD. However, participation from all six states in the STORM network may eventually broaden the reach particularly since clinical experts from each state will be presenting didactics and cases. Another potential limitation is the concern about medicolegal liability for both parties. It is important to note that the Project ECHO methodology is not telemedicine, which is to provide shared legal responsibility of clinical care for a primary patient, but instead a telementoring approach to connect, teach, and empower PCPs to become more knowledgeable about how to deliver evidence-based care to their existing patients, and to increase their confidence and capacity to expand their practice.

We have designed and replicated Project ECHO to improve provider competence in managing children and young adults with SCD. Through STORM TeleECHO, we will disseminate the NHLBI evidence-based guidelines with the goal of increasing equitable care and improving clinical outcomes. This low-cost technology-enhanced CME intervention has potential for replication in other pediatric chronic illnesses populations facing health disparities including lack of access to high-quality evidence-based care. Planned program assessments guided by the conceptual framework of an approach to continuous planning and assessment in CME will determine its utility for improving uptake and care for children and adults with SCD.
